# Surface Passivation of Silicon Using HfO_2_ Thin Films Deposited by Remote Plasma Atomic Layer Deposition System

**DOI:** 10.1186/s11671-017-2098-5

**Published:** 2017-05-04

**Authors:** Xiao-Ying Zhang, Chia-Hsun Hsu, Shui-Yang Lien, Song-Yan Chen, Wei Huang, Chih-Hsiang Yang, Chung-Yuan Kung, Wen-Zhang Zhu, Fei-Bing Xiong, Xian-Guo Meng

**Affiliations:** 1 School of Opto-electronic and Communication Engineering, Fujian Key Laboratory of Optoelectronic Technology and Devices, Xiamen University of Technology, Xiamen, 361024 China; 2 Department of Electrical Engineering, Da-Yeh University, ChungHua, 51591 Taiwan; 3 Department of Physics, OSED, Xiamen University, Xiamen, 361005 China; 4 Department of Electrical Engineering, National Chung-Hsing University, Taichung, 40227 Taiwan

**Keywords:** HfO_2_ thin films, Atomic layer deposition, O_2_ plasma pretreatment, Surface passivation

## Abstract

Hafnium oxide (HfO_2_) thin films have attracted much attention owing to their usefulness in equivalent oxide thickness scaling in microelectronics, which arises from their high dielectric constant and thermodynamic stability with silicon. However, the surface passivation properties of such films, particularly on crystalline silicon (c-Si), have rarely been reported upon. In this study, the HfO_2_ thin films were deposited on c-Si substrates with and without oxygen plasma pretreatments, using a remote plasma atomic layer deposition system. Post-annealing was performed using a rapid thermal processing system at different temperatures in N_2_ ambient for 10 min. The effects of oxygen plasma pretreatment and post-annealing on the properties of the HfO_2_ thin films were investigated. They indicate that the in situ remote plasma pretreatment of Si substrate can result in the formation of better SiO_2_, resulting in a better chemical passivation. The deposited HfO_2_ thin films with oxygen plasma pretreatment and post-annealing at 500 °C for 10 min were effective in improving the lifetime of c-Si (original lifetime of 1 μs) to up to 67 μs.

## Background

High-quality surface passivation is very important for a range of crystalline silicon (c-Si)-based electronic devices, and especially for high-efficiency c-Si solar cells. As the need for lower-cost silicon solar cells increases, since Si material has a rather high cost, thinner Si substrates are required. Therefore, their surface/volume ratio of such substrates and the contribution of their surfaces to the overall performance are increasing. Traditional surface passivation for Si involves the formation of a thin silicon dioxide (SiO_2_) layer. However, this process requires a high thermal budget process, which involves long period at high temperature. Owing to these process-related issues, considerable efforts have been made in the past to develop low-temperature surface passivation methods for both heavily doped and moderately doped c-Si surfaces. Besides SiO_2_, other layers such as SiC, a-Si:H and Si_3_N_4_ have been used for surface passivation [1]. Recently, Al_2_O_3_ films that are grown by atomic layer deposition (ALD) have been demonstrated to provide good surface passivation on c-Si [2–4]. ALD technique is a powerful method. It provides a high-level degree of precise control over the properties of the material, and especially the morphology and thickness of dielectric layers.

In the advanced semiconductor industry, hafnium dioxide (HfO_2_) thin films are used to replace SiO_2_ as the gate dielectric in field-effect transistors because they have better functionality and performance at lower cost [5, 6]. Additionally, the high refractive index of HfO_2_ makes it a potential candidate for anti-reflection coatings [7] and interference filters [8]. However, its surface passivation properties, particularly on c-Si, have scantly been studied. For example, Jun Wang et al. [9] presented the surface passivation properties of a Si surface using a thin HfO_2_ layer grown by ALD without further annealing. In another study Huijuan Geng et al. [10] reported advanced passivation using simple materials (Al_2_O_3_, HfO_2_) and their compounds H_(Hf)_A_(Al)_O deposited by ALD. All of the previous attempts were performed to deposit HfO_2_ on c-Si substrates without any pre-treatments.

In this work, the surface passivation properties of the HfO_2_ films deposited by a remote plasma atomic layer deposition system (RP-ALD) on p-type c-Si with and without in situ oxygen plasma pretreatment were investigated. Samples were annealed at different temperatures by rapid thermal annealing (RTA) system. The structural changes and the electrical properties of the thin films induced by RTA were characterized by field-emission transmission electron microscope (FE-TEM), X-ray photoelectron spectroscopy (XPS) and capacitance-voltage (C-V) measurements. The passivation mechanism of HfO_2_ films on Si is also investigated.

## Methods

In this study, (100) oriented boron-doped p-type crystalline Czochralski (Cz) Si wafers that were polished on both sides and had a resistivity of 30 Ω · cm, original lifetime of 1 μs and a thickness of 250 μm were used. Prior to the deposition of the HfO_2_ film, all wafers were cleaned through a standard Radio Corporation of America (RCA) cleaning process followed by a dip in diluted hydrofluoric acid (HF) solution (5%) for 2 min to remove the native oxide and dried in nitrogen.

The HfO_2_ thin films were grown in an RP-ALD reactor (Model: Picosun, Finland) using tetrakis (ethylmethylamino) hafnium (TEMAH) and remote O_2_ plasma as the precursors for hafnium and oxygen respectively with N_2_ as the carrier gas. In the ALD process, one deposition cycle consisted of two half cycles, one TEMAH pulse (for 1.6 s) and one O_2_ plasma pulse (for 10 s). The nitrogen purge times for TEMAH and O_2_ were 10.0 and 12.0 s, respectively. The samples were divided into two groups. For group one, HfO_2_ thin films were deposited directly on the cleaned Si wafers. For group two, before deposition of HfO_2_ thin films, Si wafers were additionally treated by remote O_2_ plasma for 1 min. The O_2_ plasma power for the pretreatment and for the ALD deposition process was 2500 W. The HfO_2_ films for all of the samples were deposited at 250 °C. Different HfO_2_ thickness (5, 15, and 25 nm) were prepared on as-cleaned Si wafers followed by annealing at 500 °C, and the corresponding minority carrier lifetimes of the passivated wafer were 9.98, 66.8, and 4.2 μs, respectively, at the injection level of 3 × 10^14^ cm^−3^. Therefore, the thickness of 15 nm (corresponding to 168 ALD cycles) was used. The substrate pre-treatment could affect nucleation, leading to different film thickness. The thicknesses of the deposited HfO_2_ are 15 nm ± 0.5 nm and 13 nm ± 0.7 nm for the samples with and without the oxygen plasma pretreatment, respectively. The wafer was flat on a platen. The double side coated samples processed twice, with a break in vacuum to flip the wafer in the chamber. The HfO_2_ thin films were deposited on 2-in wafers. As the substrate holder was about 8 in, four samples were placed on the holder and processed at a time. The samples in the two groups are referred hereafter as SD (direct depositing samples) and SO (O_2_ plasma pretreatment samples), respectively. Annealing process was performed using a RTA system at 400–650 °C in N_2_ ambient for 10 min. Samples were identified with suffixes A400 to A650 that represent the annealing temperatures. Table 1 lists the samples.

**Table 1:** Details of the HfO_2_ thin films

Sample	O_2_ plasma pretreatment	Annealing temperature(°C)
SD	N/A	N/A
SD-A400	N/A	400
SD-A450	N/A	450
SD-A500	N/A	500
SD-A550	N/A	550
SD-A600	N/A	600
SD-A650	N/A	650
SO	Yes	N/A
SO-A400	Yes	400
SO-A450	Yes	450
SO-A500	Yes	500
SO-A550	Yes	550
SO-A600	Yes	600
SO-A650	Yes	650

The minority carrier lifetimes (*τ*_eff_) of the samples were assessed by photo-conductance decay method (Model: WCT-120, Sinton lifetime tester) in the quasi-steady state mode. Metal-insulator-semiconductor (MIS) structures were prepared by depositing Al electrodes with diameters of 500 μm onto the passivation layer using a sputter system and a shadow mask. The C-V characteristics were measured with a HP4284A semiconductor characterization system to extract the electrical parameters. The chemical composition and states of elements in the HfO_2_/Si were analyzed by XPS (Thermo Fisher K-Alpha). The ion energy used for the depth profile was 3000 eV. The physical thicknesses, microstructure and interface properties of the HfO_2_ thin films were determined by FE-TEM (JEM-2100 F).

## Results and Discussion

**Fig. 1:**
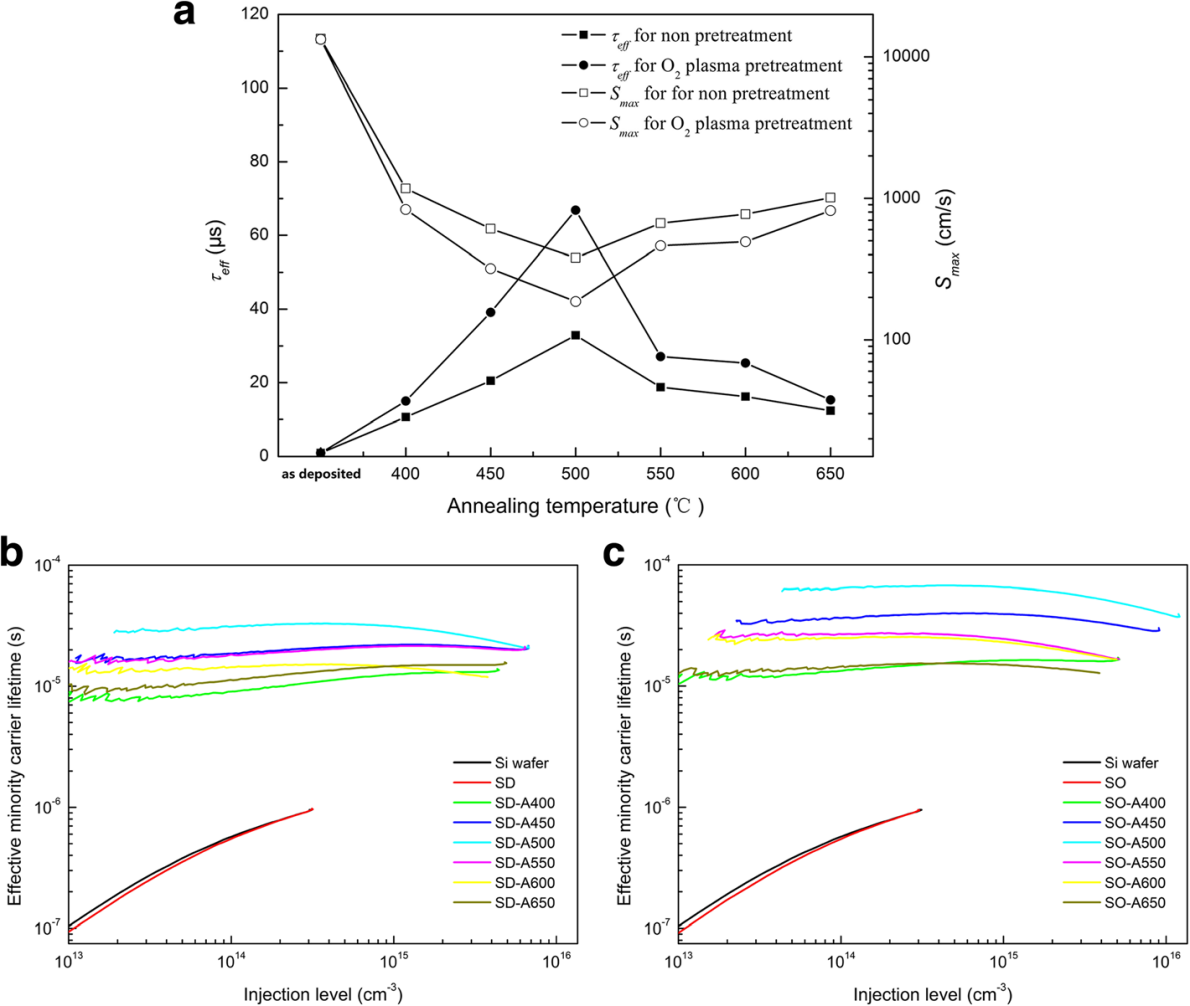
**a** *τ*_eff_ and *S*_*max*_ of the samples at the injection level of 3 × 10^14^ cm^−3^. Injection level-dependent effective minority carrier lifetime of the (**b**) SD and (**c**) SO samples

Generally, the quality of passivation is assessed in terms of *τ*_eff_ or surface recombination velocity (SRV = *S*_*max*_). The *τ*_eff_ refers to the recombination at surface defects. Figure 1(a) plots *τ*_eff_ and *S*_*max*_ for all samples at the injection level of 3 × 10^14^ cm^−3^. The *τ*_eff_ measurements were performed three times for each sample in the different locations, and the errors of the minority carrier lifetime were within ±5%. As the annealing temperature was increased from 400 to 500 °C, the *τ*_eff_ of the annealed HfO_2_ sample with O_2_ plasma pretreatment at the injection level of 3 × 10^14^ cm^−3^ increased significantly. The increase of the annealed HfO_2_ samples without O_2_ plasma pretreatment at the same injection level was much less than that of the annealed HfO_2_ samples with O_2_ plasma pretreatment. At lower temperatures (*T* < 500 °C), the annealed HfO_2_ samples without O_2_ plasma pretreatment had lower *τ*_eff_ than those with O_2_ plasma pretreatment. The annealing process provides energy to the HfO_2_ layer to active the passivation. When the annealing temperature higher than 500 °C, the minority carrier lifetime decreases, which might be due to the defects generated by the increased microcrystalline fraction and grain boundaries in the HfO_2_ layer. The O_2_ plasma pretreatment sample that had been annealed at the temperature of 500 °C had the highest *τ*_eff_ of 67 μs, corresponding to an *S*_*max*_ value of 187 cm/s. This calculation was based on the quasi steady-state photo conductance (QSSPC) *τ*_eff_ data for the injection level of 3 × 10^14^ cm^−3^. *S*_*max*_ represents the upper limit of SRV, and is estimated from the measured lifetime values using the following relation [11],

1 $$ {S}_{max}=\frac{W}{2{\tau}_{eff}}, $$

where *W* (=250 μm) is the thickness of the silicon substrate. The lower value of *S*_*max*_ can be attributed to a lower density of interface traps. It also can be seen from Fig. 1a that the O_2_ plasma pretreatment samples exhibited better passivation than the directly deposited samples, so they had a lower interface recombination velocity. This difference is attributable to the diffusion of O from the O_2_ plasma to the interfacial region to form a SiO_2_ thin film, which provides better chemical passivation of the dangling bonds.

Figure 1b, c shows the injection level-dependent effective minority carrier lifetime of the samples without and with O_2_ plasma pretreatment. For the SO samples, the minority carrier lifetime increases with the annealing temperature between 400 and 500 °C. All of the SO samples without annealing exhibited almost no passivation, and their *τ*_eff_ values were similar to that of the bare Si wafer. However, *τ*_eff_ of the annealed samples increased significantly and then decreased as the injection level increased from 4 × 10^13^ cm^−3^ to 5 × 10^15^ cm^−3^. The drop in *τ*_eff_ with increasing injection levels is caused by Auger recombination in the bulk of the c-Si substrate. The *τ*_eff_ of the as-deposited samples depends very strongly on injection level, decreasing by approximately one order of magnitude as the injection level is decreased from 3 × 10^14^ to 10^13^ cm^−3^. This dependence in injection levels is much weaker for the annealed samples. The *τ*_eff_ values of the annealed samples decrease only slightly as the injection level is reduced [12].

**Fig. 2:**
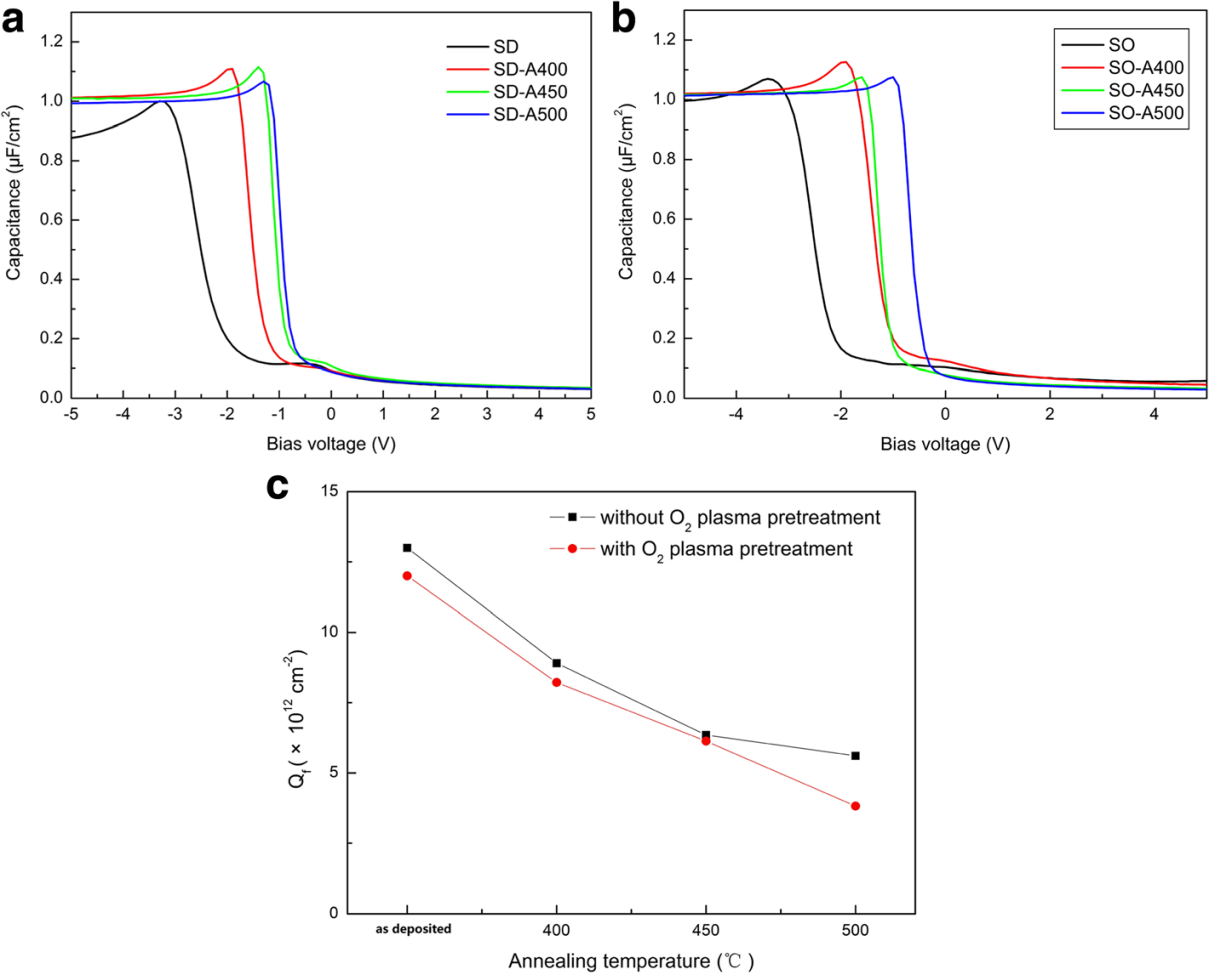
C-V characteristics measured at 1 MHz for (**a**) directly deposited samples without O_2_ plasma pretreatment, and (**b**) samples with O_2_ plasma pretreatment; (**c**) estimated *Q*_*f*_ of the annealed HfO_2_ thin films

C-V measurements are commonly used to characterize the quality of dielectric layers and their interface with the substrates. C-V measurements were performed herein at room temperature in the dark conditions at 1 MHz on a standard MIS (Al/HfO_2_/p-Si) structure. Figure 2a, b shows the C-V curves of the HfO_2_ thin films without and with O_2_ plasma pretreatment, respectively. The voltage (*V*_*A*_) that was applied across the MIS device was varied (-5 V < *V*_*A*_ < 5 V) with a sweep step length of 100 mV and signal amplitude of 50 mA, shifting from accumulation to inversion. The shift of C-V curves toward negative voltages demonstrates the presence of effective oxide charges of positive polarity in the as-deposited HfO_2_ thin films. The effective oxide charge represents the sum of mobile ionic charges (*Q*_*m*_), oxide trapped charges (*Q*_*OT*_) and oxide fixed charges (*Q*_*f*_). *Q*_*f*_ significantly affects the flat band voltage (*V*_*FB*_), as it is located at the oxide-semiconductor interface. In Fig. 2a, the C-V curves are shifted in the positive direction by the *V*_*FB*_ shift because *Q*_*f*_ decreases as the annealing temperature increases. The slope of the C-V curve increases with the annealing temperature increases, indicating that the interface trap density decreases as the annealing temperature increases. The HfO_2_ thin films with O_2_ plasma pretreatment exhibited a similar relationship, as shown in Fig. 2b. The presence of fixed charges arose from the charged oxygen vacancies in the films [13]. The fixed charge density is estimated using Eq. (2), assuming a negligible effect of the interface traps [14],

2 $$ {V}_{FB}={\phi}_{ms}-\frac{q{ Q}_f}{C_{ox}}, $$

where *ϕ*_*ms*_ (=0.32 eV)*, q* (=1.602 × 10^−19^ C), *C*_*ox*_, and *V*_*FB*_ are the difference between the work functions of metal and the semiconductor, the electronic charge, the capacitance of the dielectric per unit area and the flat band voltage, respectively.

The values of *Q*_*f*_ for the as-deposited and annealed HfO_2_ thin films are shown in Fig. 2c. *Q*_*f*_ decreases as the annealing temperature increases. The annealing process appears to reduce the density of oxygen vacancies that are responsible for the presence of positive fixed charges, which may be related to the reconstruction of the oxide film near the interface [15]. Furthermore, the *Q*_*f*_ of SO samples are lower than that of SD samples at the same annealing temperature. The interfacial defect density (*D*_*it*_) is determined using an approximation method given by W. A. Hill and C. C. Coleman [16]. The *Q*_*f*_ and *D*_*it*_ values are listed in Table 2.

**Table 2:** Calculated fixed charge density (*Q*_*f*_) and interface defect density (*D*_*it*_) from C-V measurement of the HfO_2_ thin films

Sample	Fixed charge density, *Q*_*f*_ (×10^12^ cm^−2^)	Interface defect density, *D*_*it*_ (×10^13^ eV^−1^ cm^−2^)
SO	12.0	5.21
SO-A400	8.22	4.73
SO-A450	6.13	4.42
SO-A500	3.82	3.82

**Fig. 3:**
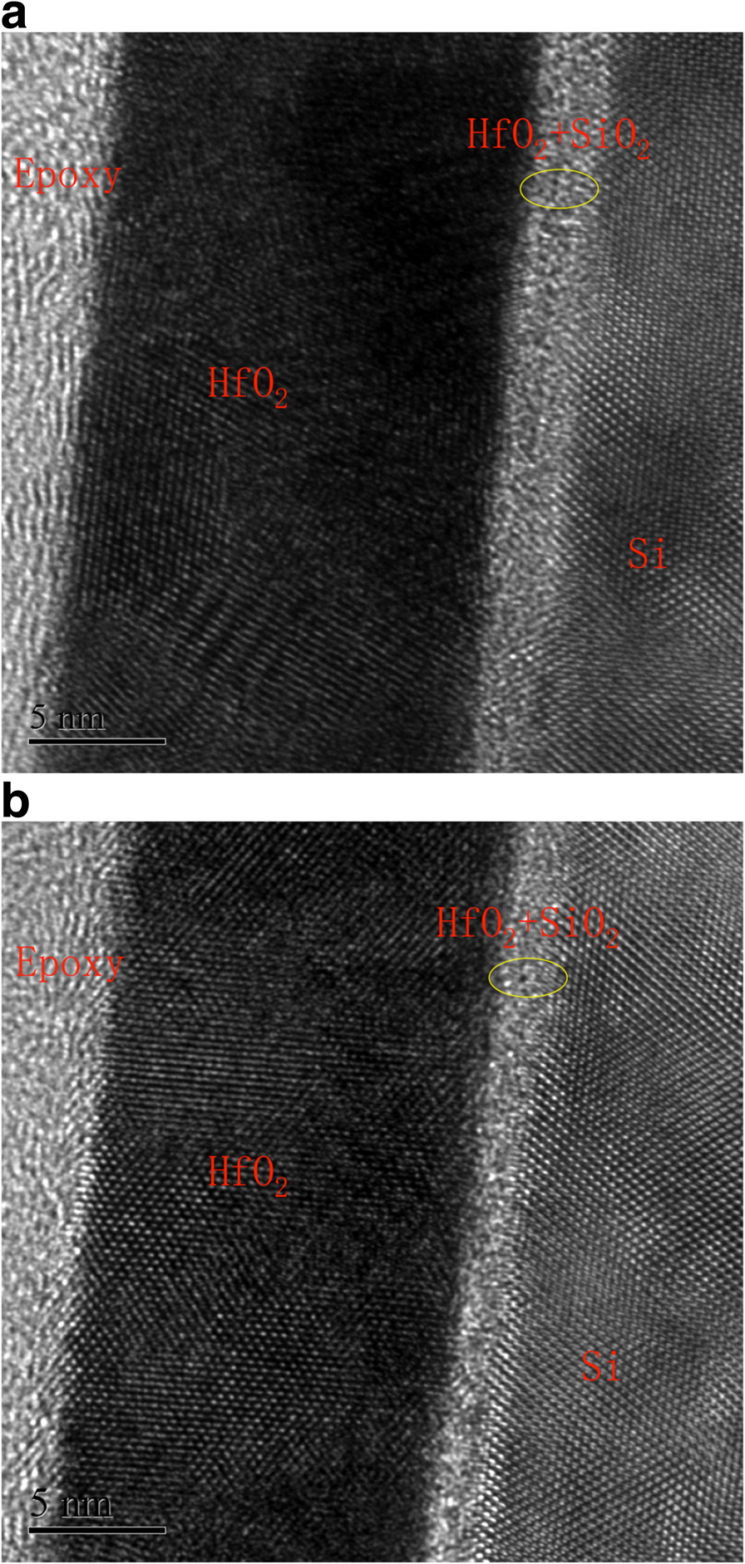
FE-TEM cross-section analysis of the HfO_2_ film annealed at 500 °C (**a**) without and (**b**) with O_2_ plasma pretreatment

Cross-sections of the annealed thin films were evaluated by a FE-TEM for assessing the film microstructure and HfO_2_/Si interface. The FE-TEM cross-section analysis of the HfO_2_ thin film annealed at 500 °C (a) without and (b) with O_2_ plasma pretreatment is shown in Fig. 3. From the FE-TEM images, the annealed HfO_2_ thin films consist of three regions, which are the HfO_2_ layer, an interfacial oxide and the Si substrate. The atoms in the HfO_2_ layer are orderly arranged in some areas, indicating that the HfO_2_ layer is microcrystalline structure. A very thin interfacial oxide layer is formed between the high *k* film and the substrate in the as deposited and annealed samples [17]. The HfO_2_ layer and the interfacial layer of the sample with oxygen plasma treatment are 15.3 and 2.7 nm, respectively. Whereas, the HfO_2_ layer and the interfacial layer of the sample without the pretreatment are 13.9 and 2.2 nm, respectively. This thickness difference should not cause the significant lifetime variation (35 and 67 μs for the samples without and with the pretreatment). Therefore, the significant lifetime improvement could be attributed to the different interface layers with the oxygen plasma pretreatment.

**Fig. 4:**
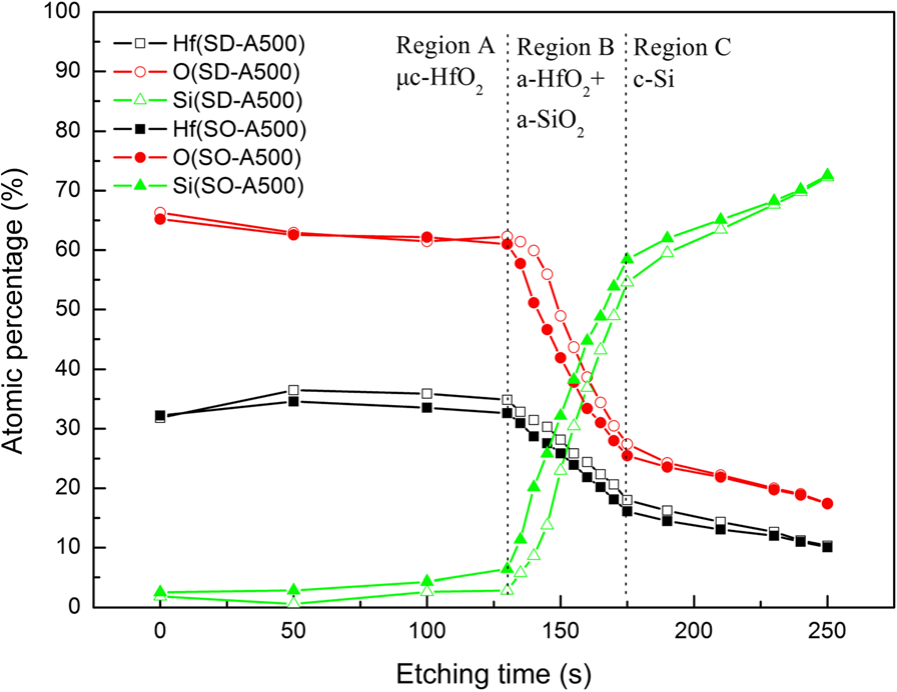
Elemental depth profiles of HfO_2_ annealed at 500 °C without and with O_2_ plasma pretreatment versus etching time

Figure 4 shows the elemental depth profiles of the HfO_2_ films annealed at 500°C without and with O_2_ plasma pretreatment obtained by XPS. Three regions are observed. In Region A, when the etching time was below 100 s, the relatively uniform atomic percentages of Hf and O corresponded to the RP-ALD μc-HfO_2_ layer. In Region B, the O and Hf atomic percentages decreased as the etching time increased from 130 to 175 s, indicating that the O elements diffused into the c-Si substrate, forming an interfacial layer [18, 19]. In Region C, when the etching time increased above 175 s, the Si signal drastically increased up to more than 60%, corresponding to the surface of the c-Si substrate. The oxygen atomic percentage and Hf atomic percentage in the c-Si substrates are due to the Ar ion sputtering effect. During the sputter process of the XPS measurement, some of the Hf or O atoms may reside on the silicon substrate surface and then be detected. Notice that in Region B, in addition to lower Hf and O with a corresponding increase in Si signal in the interface region, the sample with the oxygen pretreatment has also a larger Si signal in the bulk of the HfO_2_ film that may account for the percentage differences. The similar results can be obtained at the other investigated annealing temperatures. A possible reason might be that the O_2_ pretreatment leads to the growth of a very thin SiO_2_ layer reducing the Hf and O diffusion coming from the subsequently deposited HfO_2_. Fewer atomic vacancies are formed by diffusion in the HfO_2_ on the sample with the O_2_ pretreatment. Thus, the O_2_ pretreatment can be expected to yield fewer interface traps and exhibited higher chemical passivation quality.

Growth of a thin oxide film on a clean but unpassivated Si surface leads to the formation of new covalent bonds (chemical passivation) and termination of the dangling bonds [9]. Si/oxide interfaces often carry some fixed charges. These charges can induce an electric field at the surface of Si and can potentially reduce the recombination rate at the Si/oxide interface (field effect passivation). It has been reported by Hoex et al [20] that when preparing Al_2_O_3_ thin films by plasma ALD, they found a very thin (~1.5 nm) SiO_x_ interfacial oxide layer was formed, which provides good passivation to c-Si surface. They attributed this to the exposure of the substrate to the oxygen plasma in the very first ALD cycles. Although in this study the HfO_2_ thin films are prepared, the oxygen plasma pretreatment is found to result in a similar interfacial oxide layer (a-HfO_2_ + a-SiO_2_). The oxygen plasma pretreatment could improve the surface passivation of Si wafers.

## Conclusions

In this work, HfO_2_ thin films with a thickness of 15 nm were deposited on p-type crystalline silicon wafers using a remote plasma atomic layer deposition system. In situ remote O_2_ plasma pretreatment of the Si substrate before the deposition of HfO_2_ thin films and post-annealing at 500 °C for 10 min effectively reduced the trap density at the HfO_2_/Si interface, yielding a highest lifetime of 67 μs. The HfO_2_ thin films deposited by RP-ALD with O_2_ plasma pretreatment have potential as passivation layers in high-quality Si solar cells.

## Abbreviation

ALD: Atomic layer deposition
*C*_*ox*_: The capacitance of the dielectric per unit area
c-Si: Crystalline silicon
C-V: Capacitance-voltage
Cz: Czochralski
*D*_*it*_: Interfacial defect density
FE-TEM: Field-emission transmission electron microscope
HF: Hydrofluoric acid
HfO_2_: Hafnium oxide
MIS: Metal-insulator-semiconductor
*q*: The electronic charge
*Q*_*f*_: Oxide fixed charges
*Q*_*m*_: Mobile ionic charges
*Q*_*OT*_: Oxide trapped charges
QSSPC: Quasi steady-state photo conductance
RCA: Radio Corporation of America
RP-ALD: Remote plasma atomic layer deposition
RTA: Rapid thermal annealing
SD: Direct depositing samples
SiO_2_: Silicon dioxide
SO: O_2_ plasma pretreatment samples
SRV: Surface recombination velocity
TEMAH: Tetrakis (ethylmethylamino) hafnium
*V*_*A*_: Voltage
*V*_*FB*_: Flat band voltage
XPS: X-ray photoelectron spectroscopy
*τ*_eff_: Lifetimes
*ϕ*_*ms*_: The difference between the work functions of metal and the semiconductor
